# Dichlorido(2,9-dimethyl-1,10-phenanthroline-κ^2^ *N*,*N*′)copper(II)

**DOI:** 10.1107/S1600536809034187

**Published:** 2009-09-05

**Authors:** B. S. Wang, H. Zhong

**Affiliations:** a Department of Biochemistry, Jingdezhen Comprehesive College, Jingdezhen, Jiangxi 333000, People’s Republic of China; b The Key Laboratory of Coordination Chemistry of Jiangxi Province, Jian 343009, People’s Republic of China

## Abstract

In the title compound, [CuCl_2_(C_14_H_12_N_2_)], the complex molecule has *m* symmetry, with the mirror plane oriented parallel to the planar molecule and the ligated Cu^II^ atom. The metal centre  has a distorted tetra­hedral coordination formed by two N atoms from one 2,9-dimethyl-1,10-phenanthroline ligand and two Cl atoms. There is inter­molecular π–π stacking between adjacent 2,9-dimethyl-1,10-phenanthroline ligands, with a centroid–centroid distance of 3.733 (2)Å.

## Related literature

For backgroud to π-π stacking in metal complexes of phenanthroline and its derivatives, benzimidazole and quinoline, see: Wall *et al.* (1999 ▶); Wu *et al.* (2003 ▶); Pan & Xu (2004 ▶); Li *et al.* (2005 ▶). For bond-length data, see: Allen *et al.* (1987 ▶). 
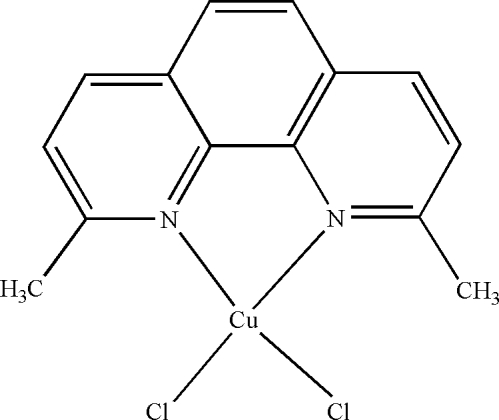


## Experimental

### 

#### Crystal data

[CuCl_2_(C_14_H_12_N_2_)]
*M* *_r_* = 342.70
Orthorhombic, 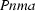

*a* = 11.239 (2) Å
*b* = 7.4651 (18) Å
*c* = 17.663 (5) Å
*V* = 1481.9 (6) Å^3^
*Z* = 4
Mo *K*α radiation
μ = 1.82 mm^−1^
*T* = 273 K
0.30 × 0.28 × 0.21 mm

#### Data collection

Bruker APEXII area-detector diffractometer
Absorption correction: multi-scan (*SADABS*; Bruker, 2000 ▶) *T*_min_ = 0.611, *T*_max_ = 0.701
10738 measured reflections
1951 independent reflections
1601 reflections with *I* > 2σ(*I*)
*R*_int_ = 0.023

#### Refinement

*R*[*F* ^2^ > 2σ(*F* ^2^)] = 0.026
*wR*(*F* ^2^) = 0.083
*S* = 1.00
1951 reflections
114 parameters
H-atom parameters constrained
Δρ_max_ = 0.41 e Å^−3^
Δρ_min_ = −0.29 e Å^−3^

### 

Data collection: *APEX2* (Bruker, 2000 ▶); cell refinement: *SAINT* (Bruker, 2000 ▶); data reduction: *SAINT*; program(s) used to solve structure: *SHELXTL* (Sheldrick, 2008 ▶); program(s) used to refine structure: *SHELXTL*; molecular graphics: *SHELXTL*; software used to prepare material for publication: *SHELXTL*.

## Supplementary Material

Crystal structure: contains datablocks global. DOI: 10.1107/S1600536809034187/at2860sup1.cif

Structure factors: contains datablocks I. DOI: 10.1107/S1600536809034187/at2860Isup2.hkl

Additional supplementary materials:  crystallographic information; 3D view; checkCIF report

## Figures and Tables

**Fig. 1.**
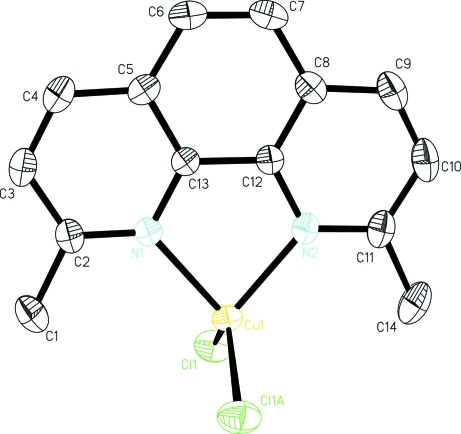
View of the molecule of (I), showing the atom-labelling scheme. Displacement ellipsoids are drawn at the 30% probability level.

**Fig. 2.**
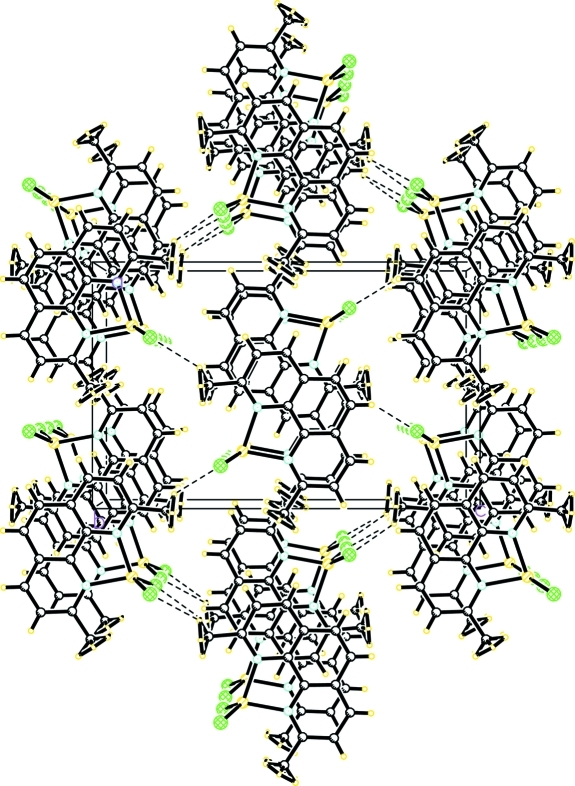
A packing diagram of (I).

## supplementary crystallographic information

### Comment

In recent years, simple metal complexes of phenanthroline and its derivatives with π-π stacking have attracted great interest because they can be used to study the hydrolysis of biologically important phosphate diesters with poor leaving groups (Wall *et al.*, 1999). A series of metal complexes incorporating different aromatic ligands such as phenanthroline(phen), benzimidazole and quinoline have been prepared and their crystal structures provide useful information about π-π stacking (Wu *et al.*, 2003; Pan & Xu, 2004; Li *et al.*, 2005). We report herein the crystal structure of the title compound, (I).

In the molecule of the title compound, (I), (Fig. 1), the bond lengths and angles (Table 1) are within normal ranges (Allen *et al.*, 1987). The two N atoms of one phen ligand and two Cl atoms are coordinated to Cu^II^ atom, in a distorted tetrahedron arrangement. The Cu—N bonds [average 2.0665 Å] are somewhat shorter than the Cu—Cl distances [average 2.1958 Å].

In the crystal structure, there is intermolecular π-π stacking between adjacent phen, with a centroid-centroid distance of 3.733 Å (symmetry code: –x, y+1/2, -z). These π-π stacking interactions lead to a supramolecular network structure (Fig. 2).

### Experimental

Crystals of the title compound were synthesized using hydrothermal method in a 23 ml Teflon-lined Parr bomb, which was then sealed. Copper chloride dihydrate (170.5 mg, 1 mmol), 2,9-Dimethyl-1,10-phenanthroline (416.5 mg, 2 mmol) and distilled water (10 g) were placed into the bomb and sealed. The bomb was then heated under autogenous pressure up to 433 K over the course of 7 d and allowed to cool at room temperature for 24 h. Upon opening the bomb, a clear colorless solution was decanted from small blue crystals. These crystals were washed with distilled water followed by ethanol, and allowed to air-dry at room temperature.

### Refinement

H atoms were positioned geometrically, with C—H = 0.93 - 0.96 Å, and constrained to ride on their parent atoms, with *U*_iso_(H) = 1.2 or 1.5*U*_eq_(C).

### Crystal data

**Table:**

[CuCl_2_(C_14_H_12_N_2_)]	*F*(000) = 692
*M**_r_* = 342.70	*D*_x_ = 1.536 Mg m^−^^3^
Orthorhombic, *P**n**m**a*	Mo *K*α radiation, λ = 0.71073 Å
Hall symbol: -P 2ac 2n	Cell parameters from 5426 reflections
*a* = 11.239 (2) Å	θ = 2.3–28.0°
*b* = 7.4651 (18) Å	µ = 1.82 mm^−^^1^
*c* = 17.663 (5) Å	*T* = 273 K
*V* = 1481.9 (6) Å^3^	Plane, blue
*Z* = 4	0.30 × 0.28 × 0.21 mm

### Data collection

**Table:**

Bruker APEXII area-detector diffractometer	1951 independent reflections
Radiation source: fine-focus sealed tube	1601 reflections with *I* > 2σ(*I*)
graphite	*R*_int_ = 0.023
φ and ω scans	θ_max_ = 28.3°, θ_min_ = 2.2°
Absorption correction: multi-scan (*SADABS*; Bruker, 2000)	*h* = −14→14
*T*_min_ = 0.611, *T*_max_ = 0.701	*k* = −9→9
10738 measured reflections	*l* = −23→23

### Refinement

**Table:**

Refinement on *F*^2^	Primary atom site location: structure-invariant direct methods
Least-squares matrix: full	Secondary atom site location: difference Fourier map
*R*[*F*^2^ > 2σ(*F*^2^)] = 0.026	Hydrogen site location: inferred from neighbouring sites
*wR*(*F*^2^) = 0.083	H-atom parameters constrained
*S* = 1.00	*w* = 1/[σ^2^(*F*_o_^2^) + (0.0553*P*)^2^ + 0.1694*P*] where *P* = (*F*_o_^2^ + 2*F*_c_^2^)/3
1951 reflections	(Δ/σ)_max_ = 0.001
114 parameters	Δρ_max_ = 0.41 e Å^−^^3^
0 restraints	Δρ_min_ = −0.29 e Å^−^^3^

### Special details

**Table:**

Geometry. All e.s.d.'s (except the e.s.d. in the dihedral angle between two l.s. planes) are estimated using the full covariance matrix. The cell e.s.d.'s are taken into account individually in the estimation of e.s.d.'s in distances, angles and torsion angles; correlations between e.s.d.'s in cell parameters are only used when they are defined by crystal symmetry. An approximate (isotropic) treatment of cell e.s.d.'s is used for estimating e.s.d.'s involving l.s. planes.
Refinement. Refinement of *F*^2^ against ALL reflections. The weighted *R*-factor *wR* and goodness of fit *S* are based on *F*^2^, conventional *R*-factors *R* are based on *F*, with *F* set to zero for negative *F*^2^. The threshold expression of *F*^2^ > σ(*F*^2^) is used only for calculating *R*-factors(gt) *etc*. and is not relevant to the choice of reflections for refinement. *R*-factors based on *F*^2^ are statistically about twice as large as those based on *F*, and *R*- factors based on ALL data will be even larger.

### Fractional atomic coordinates and isotropic or equivalent isotropic displacement parameters (Å^2^)

**Table:**

	*x*	*y*	*z*	*U*_iso_*/*U*_eq_	Occ. (<1)
Cu1	0.76127 (2)	0.7500	0.610028 (14)	0.04178 (12)	
N1	0.79467 (16)	0.7500	0.49476 (10)	0.0418 (4)	
N2	0.58758 (17)	0.7500	0.57229 (11)	0.0445 (4)	
C1	1.0098 (2)	0.7500	0.50503 (18)	0.0684 (8)	
H1A	1.0316	0.8711	0.5170	0.103*	0.50
H1B	1.0728	0.6946	0.4767	0.103*	0.50
H1C	0.9967	0.6843	0.5510	0.103*	0.50
C13	0.6920 (2)	0.7500	0.45450 (12)	0.0406 (5)	
C11	0.4872 (3)	0.7500	0.61234 (14)	0.0558 (6)	
C2	0.8982 (2)	0.7500	0.45874 (15)	0.0517 (6)	
C12	0.58155 (19)	0.7500	0.49535 (12)	0.0403 (5)	
C14	0.4987 (3)	0.7500	0.69623 (17)	0.0803 (10)	
H14A	0.5697	0.8130	0.7105	0.120*	0.50
H14B	0.5030	0.6288	0.7142	0.120*	0.50
H14C	0.4307	0.8082	0.7182	0.120*	0.50
C10	0.3763 (2)	0.7500	0.5751 (2)	0.0711 (8)	
H10	0.3067	0.7500	0.6035	0.085*	
C8	0.4741 (2)	0.7500	0.45551 (15)	0.0500 (6)	
C7	0.4757 (3)	0.7500	0.37518 (16)	0.0624 (7)	
H7	0.4041	0.7500	0.3487	0.075*	
C5	0.6901 (2)	0.7500	0.37527 (13)	0.0502 (6)	
C6	0.5787 (3)	0.7500	0.33689 (15)	0.0610 (7)	
H6	0.5774	0.7500	0.2842	0.073*	
C9	0.3695 (2)	0.7500	0.49900 (19)	0.0664 (8)	
H9	0.2957	0.7500	0.4753	0.080*	
C3	0.9025 (3)	0.7500	0.37937 (16)	0.0676 (8)	
H3	0.9756	0.7500	0.3547	0.081*	
C4	0.8005 (3)	0.7500	0.33845 (16)	0.0687 (8)	
H4	0.8038	0.7500	0.2858	0.082*	
Cl1	0.81305 (5)	1.00503 (6)	0.66249 (3)	0.06566 (17)	

### Atomic displacement parameters (Å^2^)

**Table:**

	*U*^11^	*U*^22^	*U*^33^	*U*^12^	*U*^13^	*U*^23^
Cu1	0.04754 (18)	0.0423 (2)	0.03548 (17)	0.000	−0.00528 (11)	0.000
N1	0.0374 (9)	0.0471 (11)	0.0408 (10)	0.000	0.0006 (8)	0.000
N2	0.0450 (10)	0.0441 (11)	0.0446 (10)	0.000	0.0081 (8)	0.000
C1	0.0403 (13)	0.082 (2)	0.083 (2)	0.000	−0.0043 (13)	0.000
C13	0.0403 (11)	0.0429 (13)	0.0387 (10)	0.000	0.0002 (9)	0.000
C11	0.0560 (14)	0.0532 (16)	0.0581 (15)	0.000	0.0192 (12)	0.000
C2	0.0404 (11)	0.0570 (16)	0.0576 (14)	0.000	0.0053 (10)	0.000
C12	0.0393 (11)	0.0395 (12)	0.0421 (11)	0.000	0.0014 (9)	0.000
C14	0.094 (2)	0.090 (2)	0.0565 (16)	0.000	0.0325 (17)	0.000
C10	0.0436 (14)	0.078 (2)	0.092 (2)	0.000	0.0226 (15)	0.000
C8	0.0412 (12)	0.0492 (14)	0.0596 (14)	0.000	−0.0044 (10)	0.000
C7	0.0556 (15)	0.0730 (19)	0.0586 (15)	0.000	−0.0195 (12)	0.000
C5	0.0559 (14)	0.0558 (16)	0.0388 (11)	0.000	0.0009 (10)	0.000
C6	0.0660 (17)	0.0736 (19)	0.0434 (13)	0.000	−0.0118 (12)	0.000
C9	0.0372 (12)	0.078 (2)	0.084 (2)	0.000	0.0030 (12)	0.000
C3	0.0508 (15)	0.096 (2)	0.0560 (16)	0.000	0.0176 (12)	0.000
C4	0.0656 (17)	0.095 (3)	0.0449 (14)	0.000	0.0135 (13)	0.000
Cl1	0.0766 (3)	0.0502 (3)	0.0702 (3)	−0.0019 (2)	−0.0167 (3)	−0.0113 (2)

### Geometric parameters (Å, °)

**Table:**

Cu1—Cl1	2.1958 (6)	C12—C8	1.398 (3)
Cu1—Cl1^i^	2.1958 (6)	C14—H14A	0.9600
Cu1—N1	2.070 (2)	C14—H14B	0.9600
Cu1—N2	2.063 (2)	C14—H14C	0.9600
N1—C2	1.326 (3)	C10—C9	1.346 (5)
N1—C13	1.355 (3)	C10—H10	0.9300
N2—C11	1.331 (3)	C8—C9	1.404 (4)
N2—C12	1.361 (3)	C8—C7	1.419 (4)
C1—C2	1.498 (4)	C7—C6	1.341 (4)
C1—H1A	0.9600	C7—H7	0.9300
C1—H1B	0.9600	C5—C4	1.401 (4)
C1—H1C	0.9600	C5—C6	1.423 (4)
C13—C5	1.400 (3)	C6—H6	0.9300
C13—C12	1.436 (3)	C9—H9	0.9300
C11—C10	1.409 (4)	C3—C4	1.355 (4)
C11—C14	1.487 (4)	C3—H3	0.9300
C2—C3	1.403 (3)	C4—H4	0.9300
			
Cl1—Cu1—Cl1^i^	120.23 (3)	C11—C14—H14A	109.5
N1—Cu1—Cl1	111.53 (3)	C11—C14—H14B	109.5
N1—Cu1—Cl1^i^	111.53 (3)	H14A—C14—H14B	109.5
N2—Cu1—N1	81.60 (7)	C11—C14—H14C	109.5
N2—Cu1—Cl1	112.78 (2)	H14A—C14—H14C	109.5
N2—Cu1—Cl1^i^	112.78 (2)	H14B—C14—H14C	109.5
C2—N1—C13	119.7 (2)	C9—C10—C11	121.1 (3)
C2—N1—Cu1	129.12 (17)	C9—C10—H10	119.4
C13—N1—Cu1	111.21 (15)	C11—C10—H10	119.4
C11—N2—C12	119.2 (2)	C12—C8—C9	116.6 (2)
C11—N2—Cu1	129.05 (18)	C12—C8—C7	119.5 (2)
C12—N2—Cu1	111.71 (14)	C9—C8—C7	123.9 (2)
C2—C1—H1A	109.5	C6—C7—C8	121.0 (2)
C2—C1—H1B	109.5	C6—C7—H7	119.5
H1A—C1—H1B	109.5	C8—C7—H7	119.5
C2—C1—H1C	109.5	C13—C5—C4	116.7 (2)
H1A—C1—H1C	109.5	C13—C5—C6	119.4 (2)
H1B—C1—H1C	109.5	C4—C5—C6	123.9 (2)
N1—C13—C5	122.6 (2)	C7—C6—C5	121.3 (2)
N1—C13—C12	118.2 (2)	C7—C6—H6	119.4
C5—C13—C12	119.2 (2)	C5—C6—H6	119.4
N2—C11—C10	120.1 (2)	C10—C9—C8	119.9 (3)
N2—C11—C14	117.1 (3)	C10—C9—H9	120.0
C10—C11—C14	122.8 (3)	C8—C9—H9	120.0
N1—C2—C3	120.6 (2)	C4—C3—C2	120.3 (2)
N1—C2—C1	118.2 (2)	C4—C3—H3	119.9
C3—C2—C1	121.1 (2)	C2—C3—H3	119.9
N2—C12—C8	123.1 (2)	C3—C4—C5	120.1 (3)
N2—C12—C13	117.30 (19)	C3—C4—H4	119.9
C8—C12—C13	119.6 (2)	C5—C4—H4	119.9

Symmetry codes: (i) *x*, −*y*+3/2, *z*.
